# Role of 3′UTRs in the Translation of mRNAs Regulated by Oncogenic eIF4E—A Computational Inference

**DOI:** 10.1371/journal.pone.0004868

**Published:** 2009-03-17

**Authors:** Arti N. Santhanam, Eckart Bindewald, Vinagolu K. Rajasekhar, Ola Larsson, Nahum Sonenberg, Nancy H. Colburn, Bruce A. Shapiro

**Affiliations:** 1 Gene Regulation Section, Laboratory of Cancer Prevention, National Cancer Institute, Frederick, Maryland, United States of America; 2 Basic Research Program, SAIC-Frederick, Inc., National Cancer Institute-Frederick, Frederick, Maryland, United States of America; 3 Developmental Biology Program, Memorial Sloan Kettering Cancer Center, New York, New York, United States of America; 4 Department of Biochemistry and McGill Cancer Center, McGill University, Montreal, Quebec, Canada; 5 Center for Cancer Research, Nanobiology Program, National Cancer Institute, Frederick, Maryland, United States of America; University of California, Berkeley, United States of America

## Abstract

Eukaryotic cap-dependent mRNA translation is mediated by the initiation factor eIF4E, which binds mRNAs and stimulates efficient translation initiation. eIF4E is often overexpressed in human cancers. To elucidate the molecular signature of eIF4E target mRNAs, we analyzed sequence and structural properties of two independently derived polyribosome recruited mRNA datasets. These datasets originate from studies of mRNAs that are actively being translated in response to cells over-expressing eIF4E or cells with an activated oncogenic AKT: eIF4E signaling pathway, respectively. Comparison of eIF4E target mRNAs to mRNAs insensitive to eIF4E-regulation has revealed surprising features in mRNA secondary structure, length and microRNA-binding properties. Fold-changes (the relative change in recruitment of an mRNA to actively translating polyribosomal complexes in response to eIF4E overexpression or AKT upregulation) are positively correlated with mRNA G+C content and negatively correlated with total and 3′UTR length of the mRNAs. A machine learning approach for predicting the fold change was created. Interesting tendencies of secondary structure stability are found near the start codon and at the beginning of the 3′UTR region. Highly upregulated mRNAs show negative selection (site avoidance) for binding sites of several microRNAs. These results are consistent with the emerging model of regulation of mRNA translation through a dynamic balance between translation initiation at the 5′UTR and microRNA binding at the 3′UTR.

## Introduction

Aberrant translation of cap-dependent mRNAs encoding proteins is important for tumorigenesis and tumor progression and is frequently seen in human cancer. Deregulated signaling through the oncogenic AKT pathway results in activation of the eukaryotic translation initiation factor eIF4E. eIF4E itself has been recognized as an oncoprotein implicated in tumor cell proliferation and tumor progression in multiple cancer sites including breast and lung. Targeting of eIF4E has shown therapeutic efficacy in animal models [1] and is being explored for translation to human studies. Over-activation of translation initiation appears to act in an mRNA-specific manner [2], [3]. While many of the mRNAs preferentially regulated by eIF4E have been recently identified, the molecular signatures in these mRNAs that allow them to specifically compete for eIF4E protein binding are as yet unknown. This study is the first of its kind to use computational analysis of mRNA structure to decipher molecular signatures that identify an eIF4E target mRNA.

Specific recognition of the mRNA 5′ cap by eukaryotic initiation factor eIF4E is a rate-limiting step in translation initiation [4]. In many cancers the levels of eIF4E are elevated [5], [6]. While the consequence of this upregulation might be expected to be an increase in translation rates of all mRNAs, in actuality the increase in these eIF4E levels results in preferential increase in efficiency of translation of a select group of mRNAs encoding proteins likely to be involved in tumorigenesis and tumor progression [2], [3], [7]. Experimental evidence to explain this preferential upregulation of translation is as yet missing. It has been long known that certain mRNAs in the cell are less efficiently translated than others. These weakly translated mRNAs demonstrate deficient assembly of the eIF4F initiation complex and subsequent inefficient ribosomal loading and translation initiation and/or elongation [8]. The eIF4F complex consists of, in addition to cap binding eIF4E, a scaffold eIF4G and an RNA helicase, eIF4A [9]. Efficient eIF4F formation and unwinding of RNA secondary structure by eIF4A are important for translation initiation. While it is not readily clear why eIF4E binding to the cap structure in these weakly translated mRNAs is inefficient, some of these mRNAs appear to have an extensive 5′UTR secondary structure [10]. This observation has led to the speculation that this secondary structure in turn renders these mRNAs dependent on efficient formation of the eIF4F complex, in particular the RNA helicase activity of eIF4A [11]. In other words, under conditions of limited eIF4E, weakly translated mRNAs demonstrate weak eIF4F formation and low ribosomal loading resulting in low translation initiation. However, neither the consistent presence of complex 5′UTR structure in eIF4E target mRNAs nor the consistent absence of complex 5′UTR structure in non-target mRNAs has been demonstrated. Moreover, a similar bias in eIF4E binding affinity to 7MeGpppG cap structure has also not been demonstrated.

More recent observations implicate microRNAs (miRNAs) in the regulation of translation initiation of many of the same oncogenic mRNAs. MicroRNAs are noncoding RNAs that play a key role in regulating protein expression [12] and can act as non-coding tumor-suppressors [13], [14] or oncogenes [15]–[17]. miRNAs have been shown to interact with the translation initiation complex [18]. It is, however, not clear if the predominant mechanism of miRNA activity is through regulation of translation initiation, elongation, co-translational protein degradation, competition for the cap structure, inhibition of ribosomal subunit joining or inhibition of mRNA circularization and stabilization [2], [19], [20]. Thus, while 5′UTR structure in target mRNAs may represent one mechanism for eIF4E specificity, other mechanisms may also be important.

The present study is a novel, unbiased and comprehensive computational approach to generate molecular signatures that define eIF4E target mRNAs. Total length, 3′UTR length and G+C content emerged as predictive features, particularly when considered in combination. The analysis also identified a miRNA binding bias in eIF4E target mRNAs. These features were then tested for their ability to predict fold change (i.e., the relative change in recruitment of an mRNA to actively translating polyribosomal complexes) in response to changes in eIF4E levels. A combined classifier was also tested against a data set of mRNAs regulated at the level of translation by the AKT signaling pathway.

## Materials and Methods

We employed the two independent data sets described in some detail below. These were derived from studies in which the translational efficiency was altered by experimental manipulation of either eIF4E protein levels or AKT-signaling. The AKT data set was included to test some of the predictions obtained by analyzing the eIF4E data set. It was expected that since AKT signals through multiple molecules including eIF4E, the two data sets would show partial but not complete concurrence with each other.

### Data preparation

#### eIF4E dataset

We used the data from the study by Larsson *et al.* [3]. Briefly, immortalized human mammary epithelial cells inducibly over-expressing the translation initiation factor eIF4E were analyzed for change in translational efficiency. Each microarray experiment was performed two or three times (see the original publication Larsson *et al.* [3] for more information regarding the number of replications per group). The dataset contains 13770 mRNA identifiers annotated with a fold change in the translational efficiency. For each mRNA, fold change is defined as the relative change in recruitment of an mRNA to actively translating polyribosomal complexes in cells with or without eIF4E overexpression. Thus, fold change for a given mRNA is the ratio of [polysomal mRNA^eIF4E+^/polysomal mRNA^control^] to [total mRNA^eIF4E+^/total mRNA^control^]”. The data set was further refined to a subset of 11387 RefSeq annotated human mRNA sequences whose 3′UTR or 5′UTR were not shorter than 20 nt (Genbank). Of the 11387 Refseq mRNA sequences, we used a subset of 1835 eIF4E upregulated mRNAs and 679 downregulated mRNAs. These subsets of up- and downregulated mRNAs are based on the set of eIF4E regulated mRNAs provided in table 2 of the supplementary material of ref. [3] and they correspond to mRNAs with fold changes greater than 1.4 or smaller than 0.63, respectively and with a false discovery rate (FDR) of <10%. A set of 3814 mRNAs (a subset of the 11387 mRNA set mentioned above) with a fold change between 1.2 and 1/1.2 was used as a set of eIF4E nonregulated mRNAs.

For most of the analysis reported in this study we used the above data set. An exception was for the machine learning method. Since sequence redundancy can lead to exaggerated prediction accuracy, we eliminated redundant sequences using the program BLASTCLUST [21]. This narrowed the data set to a non-redundant subset of 9629 mRNAs, whose pairwise sequence similarity is not greater than 40% for more than 50% of the 3′UTR. From this non-redundant set, we used 4000 mRNAs as a training set and the rest, 5629 mRNAs as a test set for the machine learning method. For the compute-intensive estimation of microRNA binding site selection, we used sets of 40 highly upregulated mRNAs (fold change greater 4.0) and 1200 nonregulated mRNAs with a fold change between 1.05 and 1/1.05 for analysis.

#### AKT dataset

The AKT dataset is based on the microarray data from the study by Rajasekhar *et al.* [7] in which mouse brain progenitor cells selectively activated for AKT activity were analyzed for changes in translational efficiency. We used a set of 7496 mRNAs that have the property of having a RefSeq annotated 5′UTR and 3′UTR length of not less than 20 nt. The fold changes were computed using the gcrma protocol [22], [23] within the R Bioconductor framework [24]. As in the case of the eIF4E data (see above), for each mRNA, fold change is defined as the change of the polyribosomal RNA signal relative to the total RNA signal in cells with or without AKT overactivation.

### Sequence alignments around cap-, start- and stop-region

Ideally one would like to be able to align the profiles of all mRNAs. A general sequence/structure alignment is unrealistic due to the diversity of sequences. Therefore, we generated three non-gapped alignments of “anchor” regions relative to the cap site (20 nt), start-codon (40 nt) and stop-codon (40 nt). These alignments are used for computing a bias in sequence composition as well as probabilities of base pairing with respect to fold change. Similar non-gapped alignments around anchor regions have been used by Shabalina *et al.* [25].

### Statistics of non-parametric tests

We used standard non-parametric statistical tests calculated using the R statistics software. All correlations were computed using the Spearman correlation method [26]. Comparisons between counts of two groups having two different outcomes were obtained using the Fisher exact test [26]. Comparisons of scores of two groups were performed using the Wilcoxon-Mann-Whitney rank sum test (two-sample or one-sample test depending on whether the two groups are unpaired or paired) [26]. The Kruskal-Wallis test was used for comparisons involving more than two groups [27].

### Sequence composition bias

We developed a novel method to assess a position-specific bias of sequence-composition with respect to fold change. Assessing a bias in the median fold change of 4 groups (corresponding to the four nucleotides A,C,G,U) was performed using the Kruskal-Wallis test. We computed the Kruskal-Wallis test for the 4 groups at each position around the cap, start or stop flanking region. Each group contained the fold changes of all sequences that have a certain nucleotide at a specified position. Consequently, each alignment column was assigned a p-value, which denotes the probability that the fold changes of the four groups of sequences (according to the four possible nucleotides at the specified position) are from the same distribution.

### Secondary structure predictions using RNAplfold and RNALfold

For each individual complete mRNA, we generated secondary structure profiles using the RNAplfold program [28]. The program computes probabilities (averaged over 70 nt sequence windows) of individual pairs of bases to form base pairings. We used the maximum of these averaged probabilities with respect to one base as an estimate of the chance that this base is part of a base pair. In particular, we looked for regions that differ between up-, non- and downregulated sequences. For computing the number of stable local secondary structure elements within the entire UTR regions we used the closely related RNALfold program [29].

### Machine learning using a Support Vector Machine approach

We used the R implementation of libsvm as a library for support vector machine based machine learning [30]. As input features we used the logarithm of the total length, the logarithm of the 3′UTR length and the G+C content of the mRNA (scaled by a factor of 0.1). Using predicted microRNA binding sites as features for the machine learning algorithm did not result in an increased prediction accuracy (data not shown). The support vector machine algorithm provided by the libsvm library was not used as a two-class classifier, but instead was used to predict a real number, in this case the logarithm of the fold-change values.

### MicroRNA prediction using PITA

The microRNA–target predictions were performed using the program PITA[31]. This program predicts potential microRNA targets using an estimated free energy of binding. The secondary structure of both the microRNA and the 3′UTR of the mRNA is explicitly taken into account by computing an estimated free energy of unfolding and subtracting this unfolding energy from the binding energy [31], [32]. We chose the PITA program, because unlike other approaches (miRanda, PicTar), PITA takes explicitly the secondary structure context (i.e. 70 nt flanking regions) of the microRNA target into account. The authors of the PITA program show in their publication that the approach has for a set of 190 microRNA-target interactions an average accuracy (AUC) value of 0.79, which compares favorably with competing approaches. We used a set of 470 human microRNAs obtained from miRBase release 9.1 [33], [34] and performed PITA predictions for the 3′UTRs of the 40 upregulated and 1200 nonregulated mRNAs described in the eIF4E dataset preparation section. The considered predicted microRNA binding sites were required to have a negative free energy of binding.

We assessed the positive and negative selection pressure of a microRNA-target interaction by computing a total free energy of binding as being the sum of the free energies of binding of the different predicted binding sites of the microRNA and the mRNA 3′UTR sequence. The total free energy of binding of a microRNA-target pair was compared with the total free energy of binding of that microRNA with randomly shuffled versions of the mRNA 3′UTR sequence. We created a program ALIGNEDIT that, among other things, can randomly shuffle nucleotide sequences while preserving their dinucleotide content (this program is available as part of the KNetFold software [35]). The algorithm for sequence shuffling works as follows: two different mono-nucleotides of a sequence are randomly chosen and swapped. This swapping step is only accepted, if none of the 16 possible dinucleotide content values differs by more than 1% from its original value. Five rounds of shuffling are performed on each sequence where each round ensures that each nucleotide position is subject to at least one swapping operation.

We computed for each microRNA-mRNA pair a z-score from the total free energy of binding of the microRNA and the native 3′UTR sequence and the total free energies of binding of the microRNA with 20 shuffled versions of the same 3′UTR sequence. The z-score for each microRNA was used to compute a Fisher exact test based on the counts of positive and negative z-scores in upregulated or nonregulated mRNAs. Since the total free binding energy of a 3′UTR was compared with its dinucleotide shuffled counterparts, the results obtained did not need to be normalized or corrected for length or G+C content effects.

This methodology is similar but not identical to the approach described by Stark *et al.* [36], where it is demonstrated that the often overlooked negative selection of microRNA-target interactions (the avoidance of microRNA binding sites on the 3′UTR) can provide important insights into microRNA function. Note that comparing total free energies of binding can pick up smaller differences (e.g. strength of binding) between two microRNA-target sequence pairs than simply comparing counts of predicted binding sites.

## Results

### Region specific sequence composition influences eIF4E responsiveness

**Figure 1:**
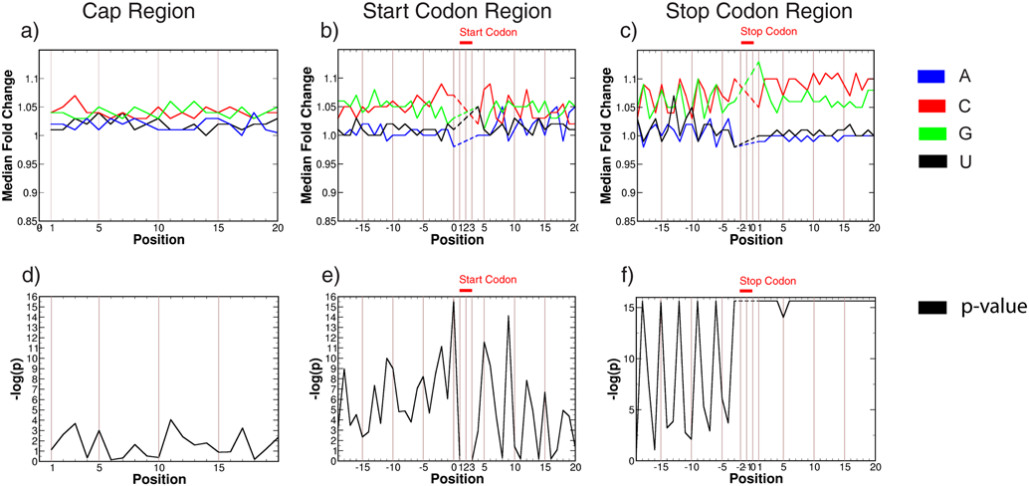
Sequence composition influences eIF4E responsiveness. Top row: median fold change of four groups of sequences corresponding to the four possible nucleotides at each position in the alignment around a) cap region (nt 1–20), b) start region (positions −19…20 with position 1 being the first nt of the coding region), c) stop region (positions −19…20 with position 1 being the first nt of the 3′UTR). Blue: A, red: C, green: G, black: U. Bottom row: Negative decadic logarithm of the Kruskal-Wallis test p-value as a function of the sequence position. The statistical test is applied at each alignment column to the fold change values of the four groups mention above. Note that because the Kruskal-Wallis test is not defined for completely conserved alignment columns, the start and stop codon regions are skipped (Figures a)–c)). The eIF4E overexpression data set consisting of 11387 mRNAs was used to generate the plots (see Materials and Methods).

We applied the Kruskal-Wallis test in order to find statistically significant position-specific correlations between the nucleotide content and the observed eIF4E-dependent polyribosomal recruitment (fold change) (see Materials and Methods). The results are shown in Figures 1a–1f. Shown is the median fold change for each nucleotide at each position near the a) cap site b) start site or c) stop site. Comparison of Figures 1a) and c), reveals that at the beginning of the 3′UTR the median fold change is highest for sequences that have G or C at those positions. Near the cap region the discrepancy between fold changes of G/C versus A/U nucleotides is smaller. The p-value of the Kruskal-Wallis test indicates the importance of the nucleotide at each position (Figure 1d–1f). The periodic peaks in terms of codon triplets at the beginning and at the end of the coding region are as per previous reports [25]. This corresponds to the wobble base of a codon triplet that appears to correlate with enhanced RNA secondary structure at the third base of a codon. Regardless, at almost all positions the median fold changes are higher for sequences that have a G or C at the respective positions. The differences are largest for the beginning of the 3′UTR as well as the end of the 5′UTR followed by the coding region. The sequence composition differences are the smallest for the beginning of the 5′UTR with marginal statistical significance.

Applying the same method to the AKT data set we find roughly comparable tendencies. The corresponding p-values are, however, at only 3 positions smaller than 0.0001 (positions −18 and 19, 20 with position 1 being the first nucleotide after the stop codon), thus indicating a position-specific sequence dependence of the AKT fold change that is weaker compared to the eIF4E case (data not shown).

### Secondary structure in 5′UTR and 3′UTR shows positive correlation with eIF4E-dependent regulation

**Figure 2:**
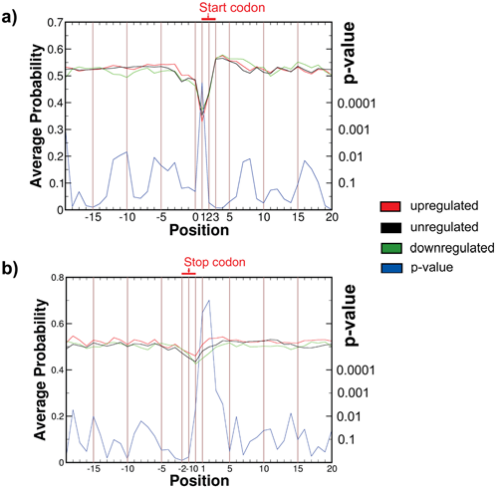
Probability of base pairing is greater for upregulated mRNAs at the regions just upstream of the start codon and flanking the stop codon. Secondary structure profiles of mRNA regions. Red, black, green: average secondary structure probability for upregulated, un-regulated or downregulated mRNAs respectively; blue: p-value of the Wilcoxon-Mann-Whitney two-sample rank sum test using logarithmic scale shown on the right y-axis. a) start codon (positions −19…20 with position 1 being the first nt of the coding region), b) stop codon (positions −19…20 with position 1 being the first nt of the 3′UTR).The positions of start and stop codon are indicated in red. The used subsets of the eIF4E overexpression data consists of 1835 upregulated mRNAs, 679 downregulated mRNAs and 3814 nonregulated mRNAs (see Material and Methods).

The base pairing probability profiles (generated using RNAplfold as described in the Materials and Methods section) of the start and stop region are shown in Figures 2a and 2b for up-, down- and nonregulated mRNAs. We used for each alignment position a Wilcoxon-Mann-Whitney two-sample rank sum test to ascertain whether the sequences of one set have on average a different probability of base pairing at that position with respect to another set. We performed three sequence set comparisons of up- versus nonregulated sequences, down- versus nonregulated sequences and up- versus down-regulated sequences. The average base pairing probabilities as well as the p-values comparing up- and down-regulated sequences for the regions around the start and the stop codons are plotted in Figures 2a and 2b, respectively.

General features of the average secondary structure of mRNAs around the start and stop codon have been observed by Shabalina *et al.* [25]. For example, on average the probability of base pairing is lower around the start and the stop codons compared to the rest of the mRNA. This can be rationalized by noting that the sequences of the start and stop-codons are under stronger evolutionary constraints. Because the start and stop codons are functionally important sequences less structure around these codons might correlate with the ribosome complex recognizing these parts of the mRNA with greater reliability.

#### 3′UTR region and stop codon

Surprisingly, the most prominent structural difference between upregulated and downregulated mRNAs is not in the 5′UTR but in 3′UTR (positions +1,+2,+3 after the stop-codon, see Figure 2b. This observation points to the 3′ UTR rather than the 5′UTR structure as a more influential factor for eIF4E-dependent regulation. At indicated positions, the probability of base pairing is on average more pronounced in the upregulated sequences compared to the downregulated sequences (p = 2.0·10^−6^, p = 2.7·10^−7^ and p = 2·10^−4^ for positions 1, 2 and 3 of the 3′UTR respectively; Wilcoxon-Mann-Whitney two-sample rank sum test). The average probability of base pairing for the nonregulated sequences around the stop codon are intermediate between those of the up- and downregulated sequence set (lower with respect to the upregulated sequences and higher with respect to the downregulated sequences). If one assumes a statistical independence between the positions of the anchored alignment, this leads to a highly significant bias towards a positive correlation between the probabilities of base pairing and the eIF4E induced shift into polysomes. At the first twenty positions of the 3′UTR region, eIF4E upregulated sequences are significantly more structured (i.e. have a higher probability of base pairing) compared to downregulated sequences (p = 1.9·10^−6^; up- versus nonregulated sequences: p = 0.02; down- versus nonregulated sequences: p = 0.0006; Wilcoxon-Mann-Whitney one-sample rank sum test).

#### 5′UTR and start codon

The average probabilities of base pairing around the start codon are shown in Figure 2a. In the 5′UTR region (indicated as positions −19 to 0) the secondary structure is at most positions more pronounced in the eIF4E upregulated than in the downregulated sequences with the nonregulated sequences being intermediate. eIF4E upregulated mRNAs are more structured at the end of the 5′UTR region (positions −19 to 0) compared to downregulated sequences (p = 0.0004, upregulated versus nonregulated sequences: p = 0.06, down- versus nonregulated sequences: p = 0.006; Wilcoxon-Mann-Whitney one-sample rank sum test). This small but statistically significant bias of eIF4E upregulated sequences towards a more stable secondary structure near the end of the 5′UTR region is consistent with a model of ribosomal scanning and unwinding of secondary structure with the eIF4A helicase [8], [37]. Interestingly, this secondary structure tendency is reversed at the first position of the start codon. At that position (corresponding to the nucleotide A in all sequences) the eIF4E upregulated sequences have on average a less pronounced secondary structure compared to the downregulated and nonregulated sequences (p = 1.8·10^−5^ and p = 9.3·10^−5^ respectively; Wilcoxon-Mann-Whitney two-sample rank sum test).

#### Coding region

Figure 2a shows (among other things) tendencies of the probabilities of base pairing corresponding to the 20 nt flanking the start codon. Similarly, Figure 2b shows the corresponding values for the 20 nt flanking the stop codon. The probabilities of base pairing show in the coding region oscillatory behavior in terms of nucleotide triplets. This was previously observed by Shabalina *et al.* [25].

The tendencies of secondary structure stability appear to be different for the beginning and the end of the coding region. At the beginning of the coding region (first 17 positions, excluding start codon), the structural differences are marginal or non significant (Figure 2a). The upregulated sequences appear to be slightly less structured than the downregulated sequences (p-value 0.02; no significant difference between up- and non-regulated sequences). At the 3′end of the coding region (Figure 2b), the upregulated sequences show for the last 17 positions (not including stop codon) higher probabilities of base pairing compared to the downregulated sequences (p = 0.005, Wilcoxon-Mann-Whitney one-sample rank sum test) and nonregulated sequences (p = 3.1·10^−5^). This bias towards more stable structure near the end of the coding region of eIF4E upregulated sequences may indicate a role for secondary structure “unwinding” activity or some other function still to be determined.

### Amount of locally stable secondary structure elements in 3′UTR regions correlates with eIF4E responsiveness

In order to analyze secondary structure content within the entire 3′UTR regions (so not be restricted to the vicinity of either cap-, start- or stop-site), we applied the local secondary structure folding program RNALfold [29]. We found a weak but significant correlation between the eIF4E fold change and the number of local secondary structure elements observed per sequence length for 3′UTR sequences (Spearman correlation; p<2.2·10^−16^, correlation coefficient 0.102). The corresponding result for the complete 5′UTR shows a weaker, but still significant correlation (p = 8.6·10^−7^, correlation coefficient 0.046). Using different energy cutoffs for counting local secondary structure elements resulted in only minor changes to the found correlations (data not shown).

### G+C content is moderately correlated with regulation by eIF4E and AKT

G+C content, especially in RNA stem structures is negatively correlated with translation efficiency (Tsien RY., RNA 2006). If the effects of eIF4E overexpression are mediated through increased eIF4F activity, then it is expected that mRNAs responsive to eIF4E levels may contain higher G+C content compared to unresponsive mRNAs.

We computed the correlation between the G+C content of mRNAs and their fold change in response to eIF4E overexpression. This was analyzed with respect to the total length (LT) of the mRNA as well as by 5′UTR (L5), coding or 3′UTR(L3) regions. These results are shown in Table 1. It is obvious that the G+C content is correlated with the eIF4E and AKT fold changes. The correlation coefficients are all positive; this means that higher G+C content is associated with a greater shift into polysomes. The correlation coefficients corresponding to total mRNA length, the 3′UTR region length and the coding region length are all positive and of comparable size. However, the correlation coefficients corresponding to the 5′UTR region are notably lower. This suggests that interactions involving the 5′UTR may be less important than previously thought in eIF4E mediated translation regulation.

**Table 1:** G+C content shows correlation with polysome shift for total mRNA, coding and 3′UTR but not for 5′UTR sequence.

	eIF4E		AKT	
	p-Value	Correlation Coefficient	p-Value	Correlation Coefficient
**Total mRNA**	<2.2·10^−16^	0.3848	<2.2·10^−16^	0.2141
**5′UTR**	<2.2·10^−16^	0.1196	8.47·10^−12^	0.0788
**Coding**	<2.2·10^−16^	0.3535	<2.2·10^−16^	0.1677
**3′UTR**	<2.2·10^−16^	0.3232	<2.2·10^−16^	0.1691

Table shows G+C content of mRNA (total mRNA or 5′UTR, coding, 3′UTR) as a function of fold change. P-values and correlation coefficients are computed according to the Spearman correlation for the eIF4E overexpression data set (11387 mRNAs) and the AKT activation data set (7496 mRNAs).

### eIF4E target mRNAs have on average shorter 3′ UTRs

We analyzed the lengths of the mRNAs with respect to fold change (the relative change in recruitment of an mRNA to actively translating polyribosomal complexes in response to eIF4E overexpression). Total length as well as 5′UTR, 3′UTR and coding region lengths were analyzed. We looked for statistically significant differences in sequence lengths with respect to eIF4E or AKT regulation. The results are shown in Table 2. Using the Spearman correlation coefficient, we computed the correlation between the length of an mRNA (total length or length of 5′UTR, coding or 3′UTR region) and its fold change with respect to the polysome shift induced by eIF4E over-expression or AKT activation. The correlations that emerged showed significant p-values (p<2.2·10^−16^), but moderate or weak correlation coefficients. For example, the eIF4E correlation coefficient was about −0.4 with respect to coding region length or total length, about −0.2 with respect to 3′ UTR length and −0.07 with respect to 5′UTR length. Since all correlation coefficients were negative, the shorter sequences are more likely to be responsive to eIF4E regulation than longer sequences. One probable explanation for this trend is that longer sequences have more alternative regulatory mechanisms (such as miRNA binding) that are associated with the mRNA translation process. The amount of eIF4E induced upregulation should therefore be lower for mRNAs with longer sequences.

**Table 2:** Length correlates with fold shift for total, coding and 3′UTR but not 5′UTR of target mRNAs.

	eIF4E		AKT	
	p-Value	Correlation-Coeff	p-Value	Correlation-Coeff
**Total length**	<2.2·10^−16^	−0.4317	<2.2·10^−16^	−0.1951
**5′UTR region**	3.07·10^−14^	−0.0711	3.36·10^−10^	−0.0725
**Coding region**	<2.2·10^−16^	−0.4138	<2.2·10^−16^	−0.1373
**3′UTR region**	<2.2·10^−16^	−0.2485	<2.2·10^−16^	−0.1463

Table of correlations between length of mRNA (total mRNA, 5′UTR, coding, 3′UTR) and the eIF4E fold change for the eIF4E dataset. P-values and correlation coefficient are computed according to the Spearman correlation for the eIF4E overexpression data set (11387 mRNAs) and the AKT activation data set (7496 mRNAs).

Length of the 3′UTR regions can be suggestive indicators of the amount of miRNA regulation of these mRNAs[38]. Upon visual inspection of the eIF4E dataset, we observed that the fold change is close to 1.0 in the extremes of 3′UTR length (smaller than 30 nt or larger than 6000 nt). One plausible explanation for this observation could be the higher degree of miRNA regulation for mRNAs with 3′UTR lengths greater than 6000 nt. Reciprocally, all mRNAs with a fold change greater than 6.0 have 3′UTR lengths of less than 900 nt. This suggests that highly upregulated genes cannot be under tight microRNA control, which in turn is related to the length of the 3′UTR regions [38].

### The Support Vector Machine shows high correlation to fold shift for combinations of total mRNA length with 3′UTR length, G+C content or both

**Figure 3:**
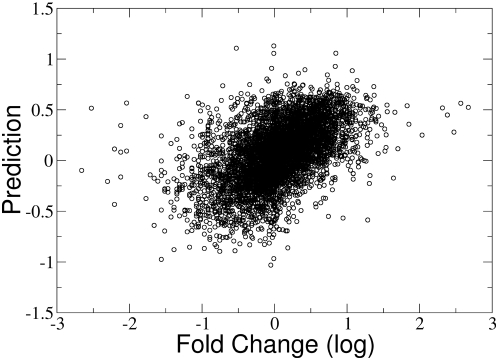
Support vector machine classifier effectively predicts fold change. Log-log plot of the eIF4E dataset fold change plotted with the corresponding support vector machine classifier results. The used eIF4E overexpression dataset consists of 4000 mRNAs for training and 5629 mRNAs for testing the classifier (see Materials and Methods).

As described in the Methods section, we used the libsvm package to train a support vector machine with features of the mRNA: total length, 3′UTR length and G+C content. The support vector machine was used to predict a real-valued number instead of a Boolean two-class classification result. Using these features, the support vector machine prediction compared with the logarithm of the fold changes yielded a Spearman correlation coefficient of 0.55 (Table 3 and Figure 3). Note that the use of the support vector machine (Table 3) leads to higher correlation coefficients compared to the results of the individual features (Tables 1 and 2). The prediction accuracy without the G+C content feature has a correlation coefficient of 0.43. Applying the support vector machine trained on the eIF4E overexpression dataset to the AKT activation dataset, we found that the support vector machine can predict the AKT fold change with a Spearman correlation coefficient of 0.15. This suggests commonalities (as well as differences) between genes differentially expressed due to eIF4E overexpression or AKT activation. 79% percent of the mRNAs that shifted in response to eIF4E were predicted, while 62% percent of those that did not shift were predicted. This result indicates a high degree of accuracy for the support vector machine (Table 4).

**Table 3:** The support vector machine shows high correlation for combinations of total length with 3′UTR length and/or G+C content.

	Spearman	Matthews
**LT+L3+GC**	0.547	0.419
**LT+GC**	0.517	0.412
**LT+L3**	0.431	0.306
**LT+L3+GC (AKT)**	0.146	0.089

Result of support vector machine. Shown is the Spearman correlation coefficient as well as the Matthews correlation coefficient of the predicted fold change versus the actual fold change using different feature combinations. LT: total length; L3: length of 3′UTR region; GC: G+C content.

**Table 4:** Confusion matrix of support vector machine for combination of total length with 3′UTR length and G+C content.

	Fold change>1	Fold change≤1
**Predicted fold change>1**	2385	1006
**Predicted fold change≤1**	618	1620

Results of the support vector machine (corresponding to first row in Table 3) applied as a two-class predictor. The used eIF4E overexpression dataset consists of 4000 mRNAs for training and 5629 mRNAs for testing the classifier (see Materials and Methods).

### Presence of miRNA binding sites is negatively correlated with eIF4E upregulation

PITA (see Materials and Methods) was used to predict mRNA – miRNA interactions using 470 human miRNA sequences available from miRBase [34]. As before, we are especially interested in differences between eIF4E upregulated and eIF4E nonregulated mRNAs. Results of the miRNA-target predictions are shown in Tables 5 and 6. Table 5 lists miRNAs whose binding sites are underrepresented in 3′UTRs of eIF4E upregulated mRNAs. Column 2 and 3 show for each miRNA the number of highly upregulated mRNAs for which the total binding energy is lower (upreg^+^) or higher (upreg ^−^) than the average of the total binding energies of the randomly shuffled sequences. Columns 4 and 5 show the corresponding information for the eIF4E nonregulated mRNAs. MicroRNA let-7i binding sites were positively selected in 6 eIF4E upregulated mRNAs and negatively selected in 34 upregulated mRNAs (negative selection in 85% of mRNAs). The corresponding counts for the eIF4E nonregulated mRNAs are 494 mRNAs with positive selection and 706 mRNAs with negative selection. This corresponds to 59% mRNAs with negative selection (p = 0.00081, Fisher exact test). The function of many of the listed miRNAs is not yet known. It is noticeable, however, that the let-7 family of miRNAs (let-7 a, b, c, d, f, g, i, mir-98 but not let-7e) is present in Table 5. Mathonnet *et al.* show, that the let-7 family of miRNAs can act as an inhibitor of translation initiation [18]. The let-7 miRNA family has also been implicated in the suppression of non-small cell lung tumor development [13] and colon cancer [13], [39].

**Table 5:** Highly upregulated mRNAs show mostly negative selection (site avoidance) for miRNA binding sites.

Name	upreg^+^	upreg^−^	nonreg^+^	nonreg^−^	p-value	ratio
miR-589	9	31	651	549	0.00008	0.41
miR-507	3	37	373	827	0.00070	0.24
let-7i	6	34	494	706	0.00082	0.36
miR-571	4	36	408	792	0.00095	0.29
miR-647	8	32	549	651	0.00109	0.44
miR-766	13	27	706	494	0.00162	0.55
miR-18b	7	33	507	693	0.00164	0.41
miR-644	9	31	572	628	0.00188	0.47
miR-622	10	30	601	599	0.00197	0.50
let-7g	7	33	487	713	0.00286	0.43
miR-363*	7	33	484	716	0.00296	0.43
miR-452*	9	31	559	641	0.00320	0.48
miR-18a	8	32	516	684	0.00326	0.47
miR-27b	10	30	585	615	0.00341	0.51
miR-550	5	35	409	791	0.00341	0.37
let-7f	7	33	481	719	0.00454	0.44
miR-98	7	33	469	731	0.00482	0.45
miR-636	9	31	532	668	0.00574	0.51
miR-502	10	30	568	632	0.00575	0.53
miR-642	13	27	658	542	0.00590	0.59
miR-330	12	28	629	571	0.00591	0.57
miR-379	4	36	346	854	0.00704	0.35
miR-30e-3p	8	32	494	706	0.00809	0.49
let-7d	8	32	493	707	0.00812	0.49
let-7b	8	32	486	714	0.00848	0.49
miR-27a	10	30	554	646	0.00922	0.54
miR-331	6	34	417	783	0.01010	0.43
miR-488	6	34	413	787	0.01019	0.44
let-7a	8	32	476	724	0.01283	0.50
let-7c	8	32	476	724	0.01283	0.50
miR-30a-3p	9	31	507	693	0.01383	0.53
miR-335	9	31	507	693	0.01383	0.53
miR-652	6	34	400	800	0.01563	0.45
miR-93	14	26	654	546	0.01588	0.64
miR-489	8	32	468	732	0.01950	0.51
miR-199a*	8	32	461	739	0.01965	0.52

List of miRNAs with negative selection (avoidance of binding sites) among 40 highly eIF4E upregulated mRNAs (fold change greater 4.0) and 1200 nonregulated mRNAs (fold change between 1.05 and 1.0/1.05). upreg^+^: number of upregulated mRNAs that show positive selection with respect to the specified miRNA, upreg^−^: number of upregulated mRNAs with negative selection, nonreg^+^: number of nonregulated mRNAs with positive selection of miRNA-target binding sites; nonreg^−^: number of nonregulated mRNAs with negative selection. P-values are computed according using a Fisher exact test, all entries with a p-value smaller than 0.02 are listed. Ratio: (upreg^+^/(upreg^+^+upreg^−^))/(nonreg^+^/(nonreg^+^+nonreg^−^)).

**Table 6:** A few upregulated mRNAs show positive selection for miRNA binding sites.

Name	upreg^+^	upreg^−^	nonreg^+^	nonreg^−^	p-value	ratio
miR-217	25	15	383	817	0.00011	1.96
miR-656	26	14	406	794	0.00013	1.92
miR-375	22	18	366	834	0.00162	1.80
miR-126*	20	20	316	884	0.00178	1.90
miR-545	25	15	459	741	0.00271	1.63
miR-607	25	15	460	740	0.00275	1.63
miR-194	26	14	483	717	0.00280	1.61
miR-374	22	18	401	799	0.00631	1.65
miR-598	22	18	405	795	0.00677	1.63
miR-220	20	20	347	853	0.00730	1.73
miR-631	22	18	413	787	0.01069	1.60
miR-325	16	24	266	934	0.01207	1.80
miR-802	24	16	474	726	0.01304	1.52

List of miRNAs with positive selection (accumulation of binding sites) among 40 highly eIF4E upregulated mRNAs (fold change greater 4.0) and 1200 nonregulated mRNAs (fold change between 1.05 and 1.0/1.05). See caption of Table 5 for an explanation of table columns.

We also identified miRNAs whose binding sites are over-represented in eIF4E upregulated mRNAs (Table 6). Upregulated mRNAs show global preferences for less miRNA binding because of their on average shorter 3′UTR length and higher G+C content. Even though the results were intrinsically normalized for 3′UTR length and nucleotide composition (because they were generated using shuffled 3′UTR sequences, see Materials and Methods), we were still able to identify a number of miRNAs that exhibit negative selection on highly upregulated mRNAs.

### Search for other sequence features

Using pattern-matching, we did find a weak correlation with the presence of the TOP motif [40] and eIF4E fold change. Sequences that start with a TOP motif have a higher median fold change (median: 1.2) than those without (median: 1.03) (p = 0.00002, Wilcoxon-Mann-Whitney two-sample rank sum test). The pattern matching was performed by filtering 5′UTR sequences with the Unix grep program and the pattern descriptor “^∧^C(C|U){4,14}(A|G)”, which indicates that a TOP motif “starts with a C residue at the cap side, which is followed by an uninterrupted stretch of 4–14 pyrimidines” [40]. Note that the definition of the TOP motif allows for variable lengths, which is not examined in typical motif search programs.

We performed analysis of 5′UTR sequences with respect to a 55mer secondary structure motif [41]. Using the program RNAMotif [42], we did not identify a correlation between the number of occurrences of the 55mer motif and the eIF4E or AKT fold change. The absence of correlation might be attributable to differences in study designs as well as cell type and cell state differences.

## Discussion

The hypothesis of this study is that inherent sequence and structural signatures in cellular mRNAs determine their sensitivity to cap-binding protein eIF4E-mediated increase in translation efficiency. This hypothesis was based on several studies demonstrating that only a select set of cellular mRNAs were preferentially recruited to actively translating polysomes as a consequence of eIF4E overexpression. While prior studies have attempted to identify these signatures, they have been limited in their approaches because either a) they analyzed only the eIF4E sensitive mRNAs and ignored the negative control i.e., eIF4E insensitive mRNAs, b) they concentrated only on either the 5′UTR or the 3′UTR of these mRNAs, or c) their datasets were smaller. Our study is the first of its kind to overcome all of the above obstacles. While our results validate some of the previous results, we were also able to identify novel molecular signatures that define eIF4E sensitive mRNAs.

Results for correlations between fold change and mRNA length as well as G+C content are similar for the AKT activation dataset and the eIF4E overexpression dataset (compare Tables 1 and 2). This is the likely explanation for why the classifier trained on the eIF4E dataset is still somewhat predictive for AKT fold change (Spearman correlation coefficient 0.23). A difference between the data sets is not entirely surprising, because AKT operates significantly up-stream compared to eIF4E on the protein interaction cascade leading to translation initiation.

### Importance of molecular signatures in the 3′UTR

Although our analysis found on average higher probabilities of RNA base pairing at the 3′ end of the 5′UTR region in the eIF4E responsive mRNAs, this effect is not as strong as we initially expected. Previous studies indicate that eIF4E mediated increased translation of select mRNAs through a 5′UTR structure[11], [43], [44].The modest influence of the 5′UTR suggests that the eIF4E sensitivity of these mRNAs may not depend solely on the efficiency of formation of the eIF4F initiation complex or on eIF4A helicase activity in particular. Thus, our results suggest that studies in the future should include both the 5′UTR and 3′UTR of an mRNA when analyzing eIF4E responsiveness.

The most striking molecular signatures of eIF4E sensitivity apparently reside in the 3′UTR region. Firstly, eIF4E upregulated mRNAs possess higher G+C content in the 3′UTR. An interpretation of that result is that a higher amount of G+C in the 3′UTR content leads to greater stability of mRNA internal secondary structure, which weakens the potential binding of translational regulatory factors such as microRNAs. Thus, a lower amount of miRNA regulation may lead to a greater response to eIF4E regulation.

This observation regarding higher G+C content is consistent with our second finding that the influence of the secondary structure on the eIF4E regulated expression is greater near the stop codon than near the start codon. The larger amount of structure in the 3′UTR may lead to less stable binding of miRNAs, resulting in reduced miRNA interference with translation. Kertesz *et al.* showed that pronounced secondary structure in the 3′UTR weakens microRNA target interactions and that microRNAs preferentially bind to the 3′UTR of the target mRNA at “accessible sites” which are less structured regions [31].

Conversely, if an mRNA is less repressed by binding microRNAs, it should be more responsive to an increased availability of the translation initiation complex. Hence, the greater the G+C content and structure in the 3′UTR region, the stronger will be the effect of eIF4E overexpression. The observation of higher probability on average of base pairing at the first three positions of the 3′UTR of upregulated sequences is to the best of our knowledge a novel finding. A probable mechanistic explanation for this observed 3′ secondary structure would be that this structure, either through a direct or indirect interaction with the ribosome, mediates a more efficient ribosome drop-off after the mRNA translation is completed. This might lead to a more efficient ribosomal recycling and enhanced translation initiation at the 5′end of the mRNA. The above scenarios are not mutually exclusive and may contribute in varying degrees to the overall phenomena. For example, the 3′UTR secondary structure may influence the binding of regulatory factors other than or in addition to microRNAs.

These findings suggest that eIF4E upregulated genes are so responsive because their 3′UTR sequences avoid binding to miRNAs that are involved in negative regulation of cell growth and proliferation. A general miRNA avoidance has been indicated in previous studies [2] in which transcripts carrying microRNA binding sites were found to be enriched among mRNAs that were translationally silenced upon eIF4E overexpression.

Binding sites for many of the microRNAs reported to have tumor suppressive effects are more likely to be under-represented in the mRNAs most sensitive to eIF4E-dependent translational upregulation. For example, upregulated mRNAs showed underrepresented binding for the tumor suppressive let-7 miRNA family and for the metastasis repressor miR-335. Another miRNA whose binding sites are underrepresented on eIF4E responsive mRNAs is miR-27b that is often decreased in breast cancer tissues and functions as a negative regulator of CYP1B1, a protein whose genotype correlates with prostate cancer risk [45]. These results lend independent support to a recent report where a global analysis of alternative 3′UTR isoforms of an mRNA in activated T cells was conducted [46]. A positive correlation between the occurrence of mRNAs with shorter 3′UTR isoforms and cell proliferation was found. This correlation was attributed to fewer miRNA binding sites in the shorter 3′UTR isoforms of an mRNA leading to higher mRNA stability and expression.

### Models for microRNA mediated translational regulation

While this manuscript was in preparation, Eulalio *et al.* [19] published an overview of miRNA mediated translational regulation. Of the 6 proposed mechanisms for microRNA-mediated translational inhibition, all involve the mRNA cap region, and 5 of the 6 mechanisms involve eIF4E specifically. This indicates more interaction between translation initiation and miRNA-mediated translational inhibition than previously anticipated. Some of the model scenarios presented in the Eulalio *et al.* review are more compatible than others with the computational results obtained in this study. All models have in common that miRNA binding has an inhibitory effect on translation initiation. Thus one expects a significant negative correlation between 3′UTR length and miRNA binding with fold change in translational activation due to eIF4E overexpression.

In addition to being compatible with our observed miRNA site exclusion and short 3′UTRs, two models discussed below would also be compatible with the observed shorter coding region and lesser influence of the 5′UTR. Model 1 postulates that miRNA-binding to the 3′UTR results in a reduced translation elongation rate. According to this scenario, eIF4E sensitive mRNAs should possess short coding regions since the inhibitory effect would be less relevant to these mRNAs. Hence, a long coding region would correspond to eIF4E nonregulated mRNAs. In addition, the correlation of the 5′UTR length with the eIF4E regulation should be much weaker or non-existent. Thus, this model is consistent with the data shown in Table 2.

In model 2, the nascent protein chain is degraded co-translationally through proteases recruited by or provided by the miRNA complex [47]. The length of the coding region could play a role: longer coding regions correspond to longer protein chains and a longer translation time, thus leading to a greater chance of a proteolytic event. This would mean that longer coding regions correspond to higher miRNA control and less eIF4E regulation. As per this model, the 5′UTR length should not be significantly correlated with translation efficiency. Our results regarding the length and fold change correlations are consistent with the contention of the model of co-translational proteolysis (Table 2). Model 1 (inhibition of translation elongation) and model 2 (co-translational protein degradation) are most consistent with the observed length dependence of eIF4E overexpression or AKT activation. It is of course possible that more than one of the microRNA-translation initiation interaction scenarios may occur in parallel or sequentially in a given cell type or physiological state.

In summary, contrary to initial expectations the 3′UTR emerges as a more significant factor than the 5′UTR in determining the eIF4E responsiveness. The eIF4E upregulated mRNAs are distinguished by shorter length of the 3′UTR and coding regions and higher G+C content of the 3′UTR. Moreover there is a statistically significant bias towards higher probabilities of RNA base pairing just upstream of the start codon and flanking the stop codon. Negative selection (site avoidance) was found for several microRNAs including the let-7 microRNA family thought to act as tumor suppressors. These results point towards several translational regulatory mechanisms acting in parallel, particularly miRNA regulation and ribosome processivity modulation by mRNA secondary structure. Clearly, the models discussed need to be experimentally evaluated. Our study has made this possible by identifying the molecular signature of the eIF4E regulated mRNAs and also by revealing the relative importance of these classifiers for eIF4E-selective response.
