# Correction: Role of 3′UTRs in the Translation of mRNAs Regulated by Oncogenic eIF4E—A Computational Inference

**DOI:** 10.1371/annotation/6a44204e-5d20-45bf-bbce-358dc9e2de27

**Published:** 2009-04-13

**Authors:** Arti N. Santhanam, Eckart Bindewald, Vinagolu K. Rajasekhar, Ola Larsson, Nahum Sonenberg, Nancy H. Colburn, Bruce A. Shapiro

The sixth author's affiliation is incorrect. It should be affiliation #1: Gene Regulation Section, Laboratory of Cancer Prevention, National Cancer Institute, Frederick, Maryland, United States of America.

